# Acceptance and Effect of Continuous Glucose Monitoring on Discharge From Hospital in Patients With Type 2 Diabetes: Open-label, Prospective, Controlled Study

**DOI:** 10.2196/35163

**Published:** 2022-05-09

**Authors:** Barbara Depczynski, Ann Poynten

**Affiliations:** 1 Prince of Wales Hospital Randwick Australia

**Keywords:** CGM, continuous glucose monitor, hospital, discharge, T2DM, type 2 diabetes, diabetes, glucose monitoring

## Abstract

**Background:**

Continuous glucose monitors (CGM) can provide detailed information on glucose excursions. There is little information on safe transitioning from hospital back to the community for patients who have had diabetes therapies adjusted in hospital and it is unclear whether newer technologies may facilitate this process.

**Objective:**

Our aim was to determine whether offering CGM on discharge would be acceptable and if CGM initiated on hospital discharge in people with type 2 diabetes (T2DM) would reduce hospital re-presentations at 1 month.

**Methods:**

This was an open-label study. Adult inpatients with T2DM, who were to be discharged home and required postdischarge glycemic stabilization, were offered usual care consisting of clinic review at 2 weeks and at 3 months. In addition to usual care, participants in the intervention arm were provided with a Libre flash glucose monitoring system (Abbott Australia). An initial run-in phase for the first 20 participants was planned, where all consenting participants were enrolled in an active arm. Subsequently, all participants were to be randomized to the active arm or usual care control group.

**Results:**

Of 237 patients screened during their hospital admission, 34 had comorbidities affecting cognition that prevented informed consent and affected their ability to learn to use the CGM device. In addition, 21 were not able to be approached as the material was only in English. Of 101 potential participants who fulfilled eligibility criteria, 19 provided consent and were enrolled. Of the 82 patients who declined to participate, 31 advised that the learning of a new task toward discharge was overwhelming or too stressful and 26 were not interested, with no other details. Due to poor recruitment, the study was terminated without entering the randomization phase to determine whether CGM could reduce readmission rate.

**Conclusions:**

These results suggest successful and equitable implementation of telemedicine programs requires that any human factors such as language, cognition, and possible disengagement be addressed. Recovery from acute illness may not be the ideal time for introduction of newer technologies or may require more novel implementation frameworks.

## Introduction

The COVID-19 pandemic has accelerated the incorporation of telemedicine including remote monitoring into patient care, including the outpatient setting [1]. There are many potential benefits associated with remote patient monitoring in a large range of chronic conditions [2]. Studies assessing the effect of telemedicine on hospital readmission rates have yielded mixed results. A capitated telehealth coaching service did not reduce hospital readmissions; however, there was a reduction in acute hospital bed days [3]. In patients with heart failure, a systematic review found that a reduction in hospitalizations was accompanied by an increase in nonemergency outpatient visits [4]. Glycemic therapy frequently requires revision during hospital stay [5]. There is little information on safe transitioning from hospital back to the community for patients who have had diabetes therapies adjusted in hospital. It is unclear whether telemedicine may facilitate this process. Unplanned hospital readmissions are higher among those with diabetes [6]. A risk factor for hospital readmission for hypoglycemia is a preceding recent hospital admission [7], suggesting that failure to adequately titrate therapy postdischarge is a contributor to readmission. Processes to facilitate better continuity of care for those with diabetes may reduce unplanned readmissions [6].

Continuous glucose monitors (CGMs) are minimally invasive, recording interstitial glucose levels every 5-15 minutes [8]. Since CGMs provide a more comprehensive overall glucose profile, both the person with diabetes and the clinician, either face-to-face or remotely, have more detailed information to personalize glycemic management plans [9]. CGMs are standard of care for most people with type 1 diabetes [10]. The role of CGMs in type 2 diabetes (T2DM) is less clear, with variable impacts on glycemic control [11-13].

The aim of our study was to determine whether the use of continuous subcutaneous glucose monitoring in patients with T2DM being discharged from hospital would reduce unplanned hospital re-presentations at 1 month as compared to a control group using capillary blood glucose meters. The secondary aim was to determine whether continuous glucose monitoring would be acceptable in this cohort.

## Methods

### Participants

The study was conducted at Prince of Wales Hospital, a tertiary referral teaching hospital in an urban area of Australia. There are approximately 50,000 admissions annually. Although servicing a broader area, the hospital is located in the Randwick local government area, where 18% of residents are aged ≥60 years and 29% of the residents are from countries where English is not their first language. Inpatient care of diabetes is primarily the responsibility of the admitting team. Consultations to the diabetes service are made on an ad hoc basis by formal referral from the admitting team. Potential participants were identified from consultations to the diabetes ward service, and so could be recruited from any medical or surgical wards. Inclusion criteria were adult inpatients with T2DM as primary or secondary diagnosis, who were to be discharged home and required postdischarge glycemic stabilization. Exclusion criteria were patients with other forms of diabetes, or who were unable to provide consent. Potential participants were approached between October 1, 2019, and March 20, 2020.

### Study Design

This was an open-label, prospective, controlled study. Potential participants were identified by the endocrinologist or fellow providing ward consultation service and recruitment was undertaken by a clinician not involved in the care of the potential participant. An initial run-in phase for the first 20 participants was planned, where all consenting participants were enrolled in an active arm. The run-in period enabled the establishment of streamlined referral pathways and familiarization with technology platforms. Subsequently, all participants were to be randomized to the active arm or usual care control group.

### Intervention

Usual care consisted of clinic review at 2 weeks (with a credentialled diabetes nurse educator who had provided the participant with education when they were an inpatient) and at 3-4 months (with an endocrinologist or trainee who had provided the inpatient diabetes consultation if new to the outpatient service, or if known to the service, then with their usual endocrinologist). In addition to usual care, participants in the intervention arm were provided with Libre flash glucose monitoring system (Abbott Australia). Education on the use of the glucose monitoring system was provided by a credentialled diabetes educator. A disposable sensor was applied to the back of the upper arm on the day of discharge. No capillary calibration is required. Participants were provided with a handheld reader and encouraged to pass the reader over the sensor at least 3 times per day. Glucose results are available in real time to the participant and glucose data can be downloaded and reviewed with the participant at the 2-week visit. The manufacturer did not supply the device and was not involved with the study in any way.

### Ethics Approval

Ethics approval was granted by the South Eastern Sydney Local Health District Human Ethics committee (18/263 HREC/19/POWH/102).

### Data Analysis

The primary outcome was to determine whether the addition of subcutaneous continuous glucose monitoring to usual care during glycemic therapy stabilization after hospital admission can reduce the number of unplanned hospital re-presentations in the first month following discharge. The secondary outcome was assessment of the acceptability of continuous glucose monitoring after hospital discharge.

We planned to recruit 440 patients. Early unplanned hospital re-presentation for patients with type 2 diabetes are up to 20% [14]. In medical service patients with glycated hemoglobin (HbA_1c_) of 8% or higher, intensification of glycemic management during admission was associated with reduced 30-day readmission (adjusted odds ratio 0.33, 95% CI 0.12-0.88) [14]. We planned to study intervention cases and controls with 1 control per intervention case. Our unpublished data for our hospital indicated that the readmission rate among controls is 20% at 1 month. If the readmission rate for experimental subjects is 10%, we will need to study 199 experimental subjects and 199 control subjects to be able to reject the null hypothesis that the failure rates for experimental and control subjects are equal with probability 0.8. The type I error probability associated with this test of this null hypothesis is 0.05. We estimated a dropout rate of 10%. Logistic regression models will be constructed for primary outcome of unplanned hospital re-presentations within 30 days of discharge with CGM provision in addition to usual care versus usual care alone as the independent variable and admission type (medical vs surgical admission) and age as covariables. To assess the secondary objective of acceptability of the CGM, the response at 2 weeks postdischarge to a 4-point Likert question (“How satisfied are you with the medical devices available for you to monitor your glucose levels?”) between the intervention and usual care groups will be compared. Data were collected prospectively.

## Results

A total of 237 patients were screened for eligibility from October 1, 2019, prior to study suspension on March 20, 2020, due to poor recruitment and restrictions on non–COVID-19 research. Overall, 136 patients were not eligible (other forms of diabetes=32, limited ability to provide informed consent due to cognitive comorbidities=34 or because the study material was only provided in English=21, antihyperglycemic agent titration was not required on discharge=14, goals of care changed to palliation=8, out of area and unable to attend for review=9, other care destination after discharge=18). Of the 101 potential participants who were approached over 5 months, 19 were recruited and completed the study. Participant characteristics are given in Table 1. Of 19 participants, 2 were readmitted within 1 month of their participation. HbA_1c_ improved (or was stable when to target) in 17 of 18 participants who had a 3-month HbA_1c_ result available. Of the 19 participants, 16 were very satisfied and 3 fairly satisfied with the medical devices available to monitor glucose levels at 2 weeks postdischarge. Of the 82 patients who declined to participate, 31 advised that the learning of a new task toward discharge was overwhelming or too stressful, 26 were not interested, 15 did not wish to attend for follow-up, 6 were approached but were discharged after trial closure, and 3 elected to self-fund CGM.

**Table 1:** Characteristics of 19 study participants.

Variable		Values
Age (years), median (range)		68.5 (32-75)
**Sex, n (%)**		
	Male	14 (74)
	Female	5 (26)
Length of stay (days), median (range)		8 (2-36)
Glycated hemoglobin at enrolment (%), median (range)		10.9 (6.5-14.8)
Glycated hemoglobin at 3 months (%), median (range)		8.0 (5-10.8)^a^
**Admission type, n (%)**		
	Endocrinology	6 (32)
	Other medical specialties	7 (37)
	Surgical specialties	6 (32)
New diagnosis of diabetes, n (%)		4 (21)
Requiring insulin on discharge, n (%)		19 (100)

^a^Result from 18 patients.

## Discussion

### Principal Findings

Only 19 of 101 potential participants were recruited to the study. Of the 19 participants in the intervention arm, only 2 (10%) had an unplanned hospital re-presentation in the first month after discharge, versus a published rate of 17% [14]. Participants were satisfied with the CGM. Although telehealth has the potential for enhanced clinical care [15], our negative study demonstrates the difficulties in implementing new technologies in a cohort of older adults with chronic illness after acute hospitalization. Our study supports previous work that suggests human factors may impede the uptake of newer technologies [16]. Our study highlights patient concerns and barriers that will need to be addressed if telemedicine is to be provided equitably. This includes addressing cognitive and mental health barriers. The provision of culturally, socially, and educationally appropriate technical material in a range of languages may be required. Acute illness and transitioning home is a stressful time for a person and their support network, and so may not be a suitable time to introduce new diabetes self-management tasks. This is in addition to limited access to technology and telemedicine “unreadiness” being high among older adults [17].

The underpowered sample size and early termination were significant limitations to our study, and we were not able to address our primary or secondary aims. A further limitation is that we were unable to ascertain whether our low recruitment rate, particularly for those not wishing to attend a follow-up clinic visit, may reflect reticence due to recovery from acute illness or chronic disease burnout. It is unclear whether our intervention would have been more acceptable if offered at a different time point in the provision of diabetes care [18]. Age may be another factor; a recent study has shown a low participation rate of approximately 10% for a remote, technology-based intervention for adults with an average age of 60 years with chronic disease [19].

### Conclusion

Diabetes therapy frequently requires adjustment on discharge from hospital. Newer technologies such as CGM provide a more comprehensive glucose profile that can be incorporated into remote patient monitoring. Where used, satisfaction with such newer technologies to facilitate telemedicine for diabetes management is high. The limited recruitment to our study suggests hospital discharge may not be the optimal time to introduce complex new technologies to patients. In embracing the promise of telemedicine including remote monitoring, further research on addressing human factors, to ensure equity, is required.

## Abbreviations

CGM: continuous glucose monitor
HbA_1c_: glycated hemoglobin
T2DM: type 2 diabetes
